# Bio-inspired broadband absorbers induced by copper nanostructures on natural leaves

**DOI:** 10.1038/s41598-020-59960-x

**Published:** 2020-02-24

**Authors:** Trung Duc Dao, Dinh Dat Pham, Thi An Hang Nguyen, Thi Viet Ha Tran, Chung Vu Hoang, Tien Thanh Pham

**Affiliations:** 1 VNU Vietnam – Japan University, Vietnam National University, Hanoi, Luu Huu Phuoc Street, My Dinh 1 Ward, Nam Tu Liem District, Hanoi 100000 Vietnam; 2 Institute of Materials Science, Vietnam Academy of Science and Technology, 18 Hoang Quoc Viet Street, Cau Giay District, Hanoi Vietnam

**Keywords:** Nanoscale materials, Metamaterials

## Abstract

In this work, two copper-based biometamaterials were engineered using leaves of water cabbage *(Pistia stratiotes)* and purple bauhinia *(Phanera purpurea)* as templates. The copper sputtering was implemented to produce a thin copper film on the surface of leaves. The scanning electron microscopy (SEM) images exhibited the root hair-like nanostructure of water cabbage leaf and single comb-like nanostructure of purple bauhinia leaf. In spite of copper coating, the leaf surfaces of water cabbage and purple bauhinia were black and exhibited excellent light absorption at visible and near infrarrred wavelengths. It was estimated that these two types of leaves could absorb roughly 90% of light. Finite-difference time-domain (FDTD) calculations predicted the low reflectance stemming from the leaf nanostructures and copper coating layer. Because of the low cost of copper as a coating metal and simple procedure, this can be a promising method for quick fabrication of a thin copper film on the leaf nanostructure for application in blackbody or as the light absorbers.

## Introduction

Metamaterials are artificial structures that can interact with electromagnetic radiation in a desired fashion^1–5^. However, there are many living creatures featuring their own forms of metamaterial structures with specific functionalities. These living things can change their colors without pigment or give hydrophobicity or bust up bacteria^5–11^. For example, those, who are lack of melanin - the pigment appears in brown eyed people, possess blue eyes. Without melanin, the blue iris stems from the structure of eyeball tissue itself not because of a different type of pigment^12^. In other words, the iris can display a natural form of metamaterial that reflects blue but selectively absorbs other colors. The compound eyes of moths *(Cameraria ohridella)* contain thousands of nanostructures on its surface, thus allowing them to see considerably better than human beings in dim and dark conditions^13^. These patterns reveal almost perfect broadband anti-reflection properties. As a result, the moth’s eye can absorb more light. A research group at Jacobs University Bremen published a paper on the design of better thin film solar cells based on nanostructured nipple arrays of the moth eye^14^. The coating that imitated the moth-eye array resulted in an increase in the short circuit current and conversion efficiency by more than 40%. For a material to be regarded as a metamaterial, it must operate at microscopic-scale and is not detectable by the naked human eyes.

The surface of lotus leaf (*Nelumbo nucifera*) has an ability to repel water strongly due to the combination of the microscale mound and the nanorod structures^15^. Recently, many studies have been conducted to fabricate biometamaterials from leaves of lotus and taro using a sputtering method and to characterize biometamaterials^16,17^. It was found that the 10 nm-thick gold biometamaterial on lotus leaf and 30 nm-thick gold biometamaterial on taro leaf demonstrated the reflectivity of lower than 0.01 through the entire visible spectrum. The low reflectivity led to near total light absorption on the material surface. Therefore, these biometamaterials could be used as the light absorbers.

**Figure 1:**
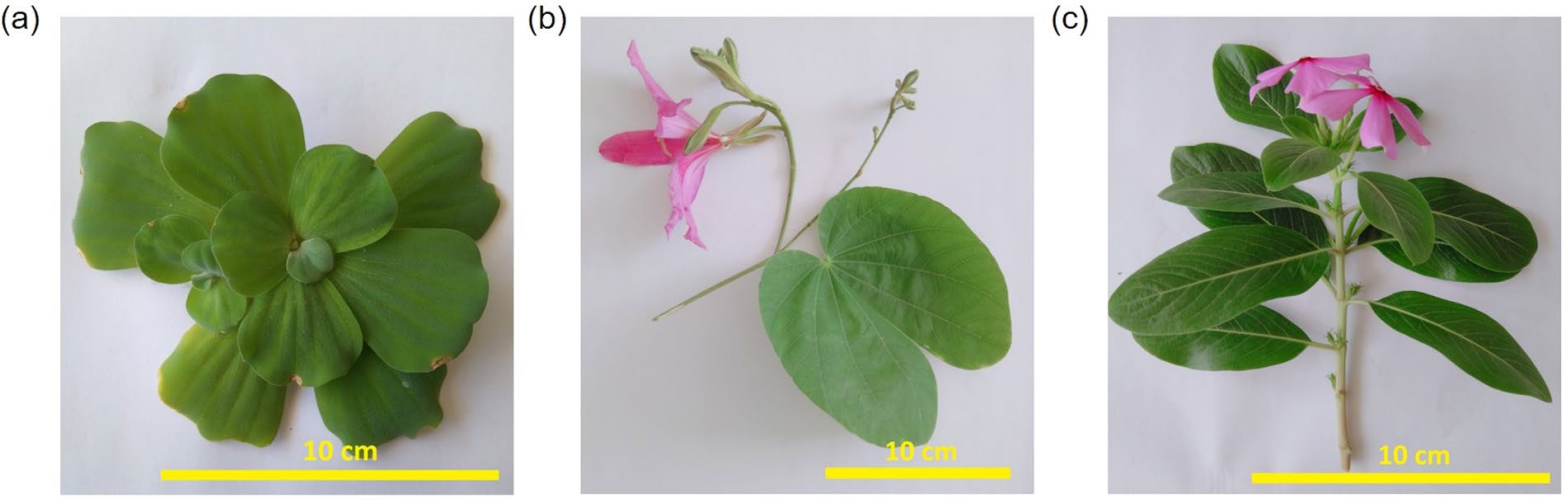
Leaf of (**a**) (bar: 10 cm) *Phanera pupurea*, (**b**) (bar: 10 cm) *Pistia stratiotes*, and (**c**) (bar: 10 cm) *Catharanthus roseus*.

*Pistia stratiotes* known as water cabbage, is an aquatic plant in the Araceae family. *Phanera purpurea* referred as purple bauhinia, is a member of the Fabaceae family. Their images are shown in Fig. 1(a,b). In this study, these two categories of leaves were used as the templates for fabrication of copper-based biometamaterials. Since the leaf of water cabbage and purple bauhinia displayed nanostructures, their leaf surfaces were distinguished by water repellence. The results of wetting performance of water for two kinds of plates are given in Fig. S1 in Supporting Information. After being coated with copper, the leaf surfaces of water cabbage and purple bauhinia were almost black. The copper coated leaf surfaces of water cabbage and purple bauhinia exhibited reflectivity of less than 2.5% through the whole visible spectrum.

## Experiments

The literature review shows that natural leaves used for fabrication of biometamaterials are normally selected based on their hydrophobicity. It is predicted that the hydrophobic leaves possess nanostructure on their surfaces. In contrast, the hydrophilic leaves are assumed to be lack of this unique structure. In the present study, the leaves of purple bauhinia (Fig. 1(a)) and water cabbage (Fig. 1(b)) represented for hydrophobic leaves, whereas the leaf of rosy periwinkle (Fig. 1(c)) exemplified hydrophilic leaves. The biometamaterials were engineered according to the following procedure: (i) The young leaves of both purple bauhinia and water cabbage were collected in the nature in Hanoi, Vietnam. (ii) After being treated with deionized water (DIW), the leaves were nipped of and fixed on a sputtering holder. (iii) A thin copper film was deposited on the leaves of the investigated plants by sputtering in argon (Ar) at a low pressure from a 99.99% copper target (supplied by Able Target Limited, China). For control, the copper coated leaves of rosy periwinkle (*Cathranthus roseus*) were prepared. The sputtering was performed using a SKE106012 (Syskey Technology Co., Ltd.) sputtering coater. The sputtering was done with a DC power of 100 W and a total pressure of about 0.13 Pa of argon (99.99% purity). The deposition was conducted at the room temperature and the time ranging from 20 to 300 s. In order to determine the thickness of copper coating layer, the copper films were also deposited onto glass substrates under the same conditions. The thickness of the copper layer on the surface of leaves was determined through the thickness of that on the glass substrates by measuring thin film step heights with the NanoMap 500LS 3D (aep Technology).

The reflection spectra were recorded with a MCPD – 3000 spectrometer (Otsuka Electronics) using halogen lamp as a light source. For reflectance measurement, the light was conveyed to samples using Y-type optical fiber at normal incidence. The silver film was used as reflectivity reference. For scattering spectra measurement, the light from light source was transmitted to samples with at normal incidence. The back-scattered light was collected by another optical fiber and transferred to the spectrometer. A SRS-99 diffused reflectance standard (Labsphere) was used as reflectance reference. The scattering angle was approximately 60° compared to the normal one.

## Results and Discussion

**Figure 2:**
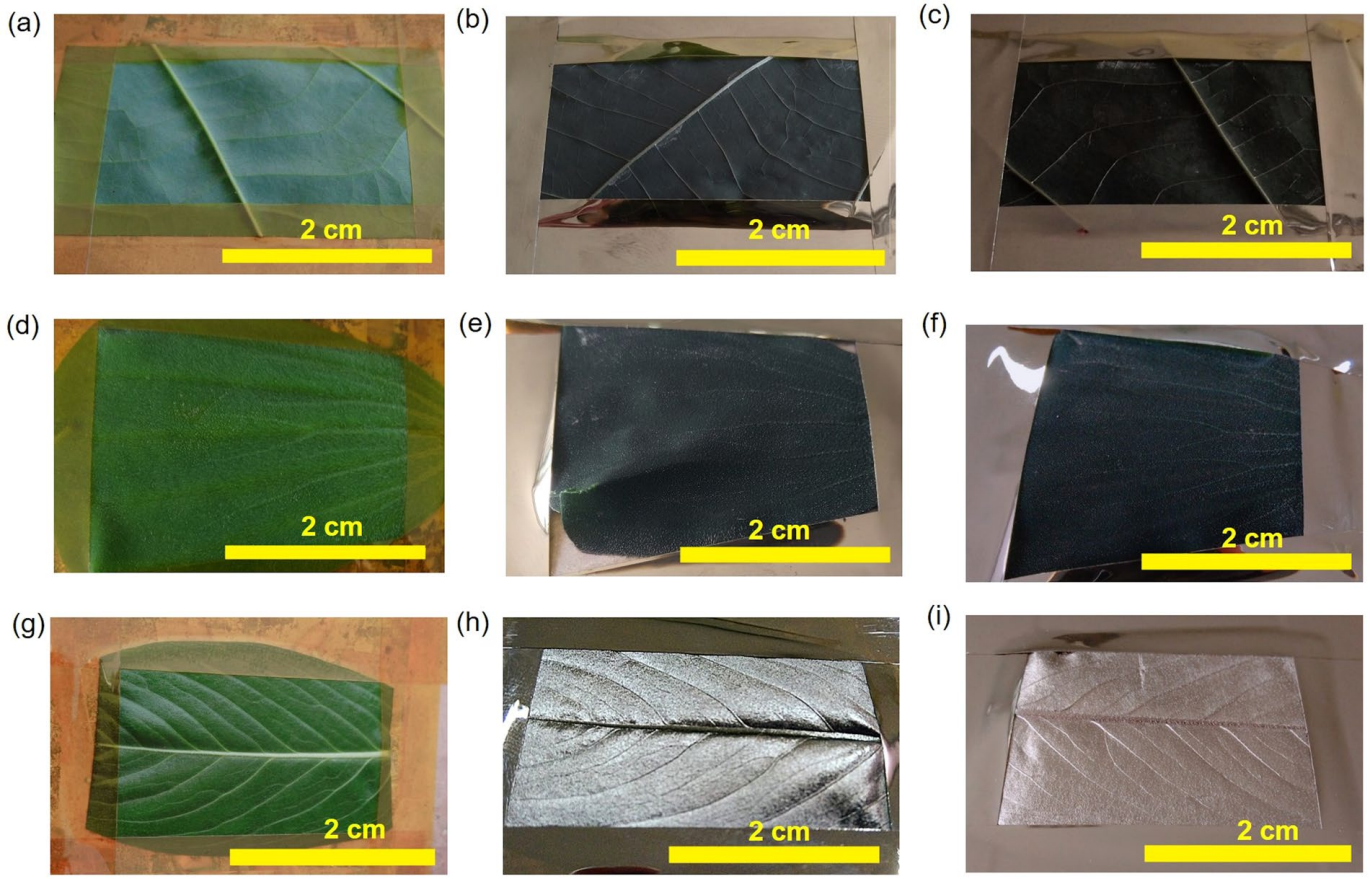
Photographs of the samples (bar: 2 cm): (**a**) *Phanera pupurea* leaf without copper films, and covered by sputtered copper films (**b**) 25-nm-thick, (**c**) 50-nm-thick, (**d**) *Pistia stratiotes* leaf without copper films, and covered by sputtered copper films (**e**) 25-nm-thick, (**f**) 50-nm-thick, (**g**) *Catharanthus roseus* leaf without copper films, and covered by sputtered copper films (**h**) 25-nm-thick, (**i**) 50-nm-thick.

Figure 2(a–i) show the images of leaves before and after coating with copper. Each row represents a different type of leaf. While the leaves of *Phanera pupurea* and *Pistia stratiotes* were employed as the object samples, the leaves of *Catharanthus roseus* were utilized as control samples. For object samples, three categories of the thickness of the copper film were applied, consisting of 0, 25, and 50 nm. The thickness of copper film was labeled as follows: (a) 0 nm, (b) 25 nm, (c) 50 nm for *Phanera pupurea* leaves, (d) 0 nm, (e) 25 nm, and (f) 50 nm for *Pistia stratiotes* leaves. For control samples, the images of (g) and (h) (i) in Fig. 2 display uncoated and coated leaves of *Catharanthus roseus*, respectively. The 0 nm copper film image corresponds to uncoated leaf, which was green and completely different from the coated leaf image.

The copper-coated leaves of *Phanera purpurea* and *Pistia stratiotes* exhibited black color and low reflectance. On the contrary, *Catharanthus roseus* displayed metal-like color with a copper film thickness of more than 15 nm. The Fig. 2(h,i) show the copper-coated leaf of *Catharanthus roseus* when the copper-film thickness was 25 and 40 nm, respectively. For the two investigated types of leaves, the surface color of the leaf coated with copper film was observed to gradually turn into black with an increase in the thickness of the copper film. The true black color was attained when the thickness of the copper film reached 20 nm. Two object leaves showed black color with the thickness of copper film in the range from 20 to 100 nm, the surface of copper coated leaf became darkest when the copper film was as thick as 50 to 60 nm. In general, the chromaticity of black color was not very sensitive to the thickness of copper film. The copper-coated leaves of *Pistia stratiotes* were always blacker than those of *Phanera purpurea*. However, when the copper film was thicker than 95 nm in case of *Phanera purpurea* and 115 nm in case of *Pistia stratiotes*, some regions on the surface of coated leaves showed the copper color. Thus, it can be concluded that the black color on the leaf surface of *Phanera purpurea* and *Pistia stratiotes* was formed only with an appropriate thickness of copper film, which was found in the range of 20–100 nm.

**Figure 3:**
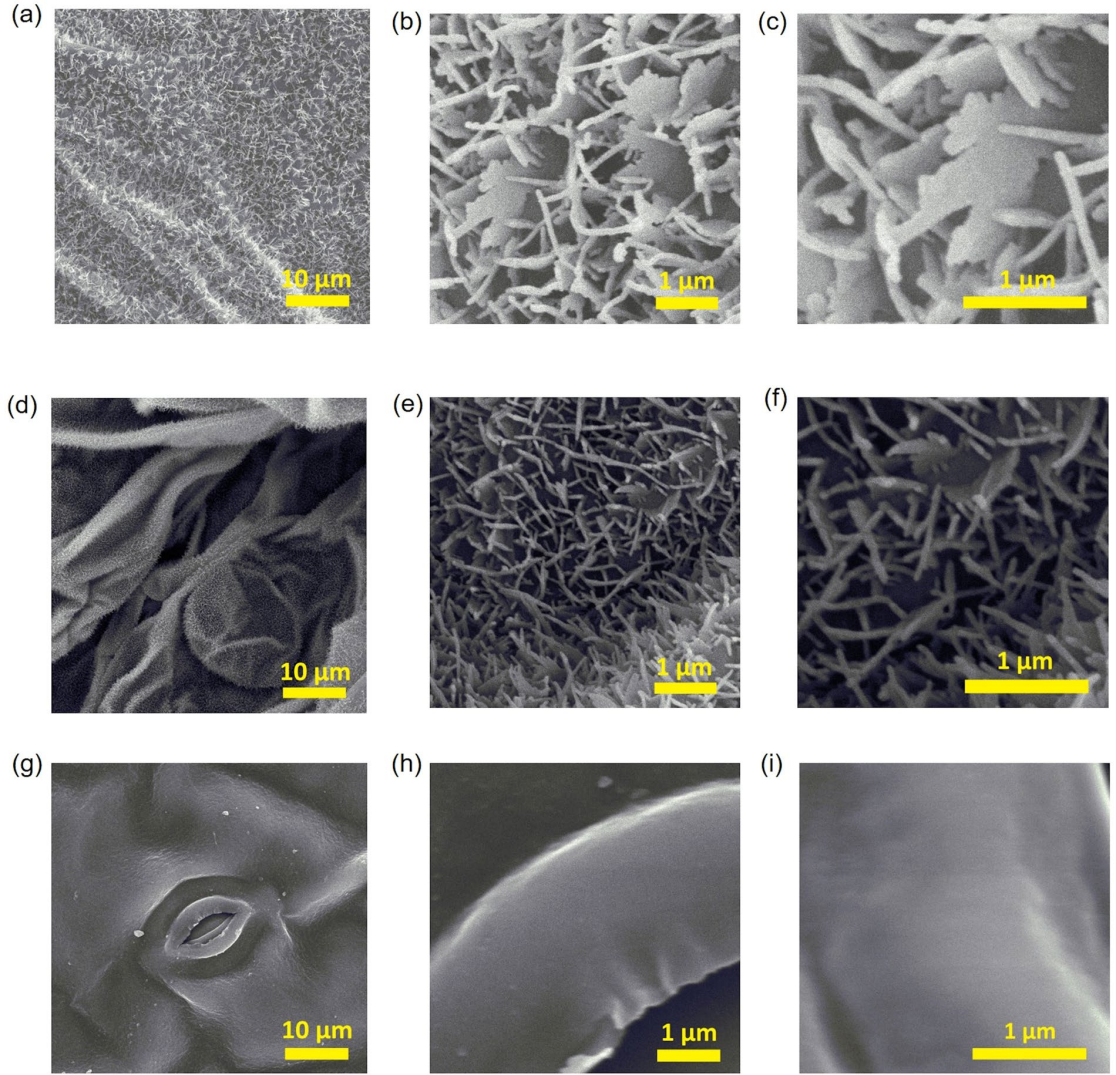
SEM images of copper-coated leaves: (**a**) (bar: 10 µm), (**b**) (bar: 1 µm), (**c**) (bar: 1 µm) magnified images of *Phanera pupurea* leaf, (**d**) (bar: 10 µm), (**e**) (bar: 1 µm), (**f**) (bar: 1 µm) magnified images of *Pistia stratiotes* leaf, (**g**) (bar: 10 µm), (**h**) (bar: 1 µm), (**i**) (bar: 1 µm) magnified images of *Catharanthus roseus* leaf.

Figure 3(a–i) show SEM images of investigated and control copper-coated leaves, which reveal the structure of the leaf surfaces. The thickness of copper film was 50 nm. It was observed that *Catharanthus roseus* leaf surface was plain. It is evident from Fig. 3(g–i) that nanoscale structure could not be observed. This explained why the color of the copper-coated leaves of *Catharanthus roseus* was similar to the copper flat film in adhesive tapes. In contrast, at the low magnification, Fig. 3(a) shows a single comb-like nanostructure of *Phanera purpurea* leaves covered with thin rectangular plates that were vertically set on edge, as shown in Fig. 3(b,c). The nanostructure on *Phanera purpurea* leaf was largely similar to that on taro leaf reported in a previous study^17^. In case of *Pistia stratiotes*, the leaf surface showed many microscale roots, which included hair-like and plate-like nanostructures with the predicted thicknesses varied from 50 to 80 nm, as shown in Fig. 3(d–f). When the thickness of copper coating layer increased, the copper covered not only the rectangular plates but also the sides, making the thickness of the rectangular plates expanded. The thickness of the plate rose from 50~80 to 120~150 nm when the thickness of the copper coating layer elevated from 50 to 130 nm. This is the reason for the change of the color of the leaf surface when varying the thickness of copper layer. The nanostructure on the leaf of both *Phanera purpurea* and *Pistia stratiotes* had random orientation and dense distribution. However, the nanostructure density of *Pistia stratiotes* leaf was substantially higher than that of *Phanera purpurea* leaf. This is why the copper coated leaves of *Pistia stratiotes* were always blacker than those of *Phanera purpurea*, as can be observed from Fig. 2.

**Figure 4:**
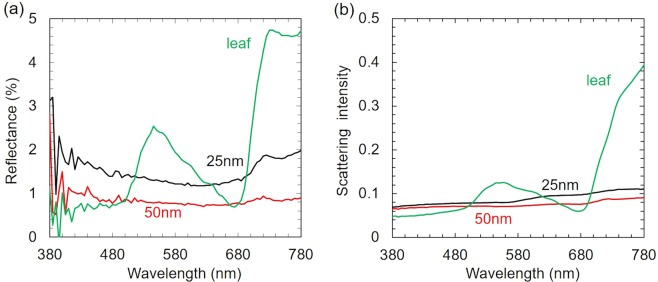
(**a**) Reflectance spectra, (**b**) Scattering spectra of *Phanera pupurea* leaf: (blue line) *Phanera pupurea* leaf without copper films, covered by sputtered copper film (black line) 25-nm-thick, and (red line) 50-nm-thick.

**Figure 5:**
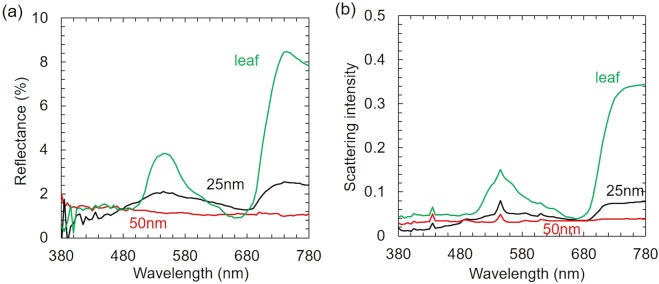
(**a**) Reflectance spectra, (**b**) Scattering spectra of *Pistia stratiotes* leaf: (blue line) *Pistia stratiotes* leaf without copper films, covered by sputtered copper film (black line) 25-nm-thick, and (red line) 50-nm-thick.

Figures 4, 5 show the reflectance and scattering spectra of *Phanera purpurea* and *Pistia stratiotes* leaves, respectively. It is evident that, the scattering efficiency of uncoated leaves was 5–10 times higher than reflectance efficiency because of the rough leaf surface. The spectra of uncoated leaves displayed two noticeable signals. The peaks at the wavelengths of about 545 nm and higher than 680 nm were due to the absorption of Chlorophyll a, b and β-Carotene in visible wavelengths (red (650–700 nm) and blue (400–500 nm)) spectra^18^, as shown in the blue line of Figs. 4(a,b) and 5(a,b). The reflectance and scattering efficiencies were found to alter with the copper film thickness. Those signals almost disappeared in spectra of copper-coated leaves, as shown in the red and black line of Figs. 4(a,b) and 5(a,b). The reflectance of copper-coated *Phanera purpurea* leaves was lower than 2%, and almost unchanged at different visible wavelengths. The copper-coated *Phanera purpurea* leaves demonstrated higher reflectance and scattering efficiencies in the absorption region of Chlorophyll and Carotene compared to the uncoated leaves. This demonstrates that the optical properties of coated leaves were not decided by the material of leaves. The 50 nm-thick copper-coated *Phanera purpurea* leaves showed the lowest reflectance in comparison with other thicknesses of this type of leaf. The lowest reflectance of this leaf was consistent with the images shown in Fig. 2(c), in which surface appeared darkest. The scattering spectra were similar to the reflectance spectra, as shown in Fig. 4(b). The scattering intensity of copper-coated *Phanera purpurea* leaves was lower than 0.07 at the entire visible spectrum, and reduced from 0.3 to 0.06 at the wavelengths of higher than 680 nm.

In case of *Pistia stratiotes* leaves, the lowest reflectance and scattering efficiencies could be achieved with the copper-film thickness of 50 to 100 nm. The reflectance spectrum and scattering intensity of the 25 nm-thick copper film showed some peaks similar to those obtained with uncoated leaves at the wavelength of about 545 nm and higher than 680 nm, as shown in the black line of Fig. 5(a,b). This may result from the storage problem with *Pistia stratiotes* samples. Few hours after fabrication, the black color of *Pistia stratiotes* gradually disappeared when the copper-film thickness was from 25 to 40 nm. However, when the copper film was thicker than 50 nm, the lower reflectance and scattering intensities of *Pistia stratiotes* leaves compared to *Phanera purpurea* leaves were observed, indicating that the former still had a high potential, as shown in the red line of Fig. 5(a,b). The reflectance and scattering intensities of 50 nm-thick copper film were lower than 2% and 0.04, respectively over the entire visible spectrum. The transmittance of these coated leaves was also found to be negligible. Thus, the absorbance was higher than 90% through the whole visible wavelength range. The extremely high absorbance obtained with *Pistia stratiotes* and *Phanera purpurea* leaves are comparable with other absorbers^16,17,19–21^. Therefore, it is possible to conclude that the copper-coated *Phanera purpurea* and *Pistia stratiotes* leaves are good candidates for being applied as the light absorbers in the visible, near infrared, and infrared wavelength regions.

The copper nanoparticles had an interbrand absorption at ~590 nm, meaning that the copper nanoparticles absorbed the light at a wavelength ranging from 380 to 590 nm. The porous nature of the *Phanera pupurea* and *Pistia stratiotes* leaves resulted in the formation of the separated copper nanoparticles in the tips or disks and the plasmon interaction of those copper nanoparticles could lead to the broad band characteristic of the absorptivity of the copper-coated *Phanera pupurea* and *Pistia stratiotes* leaves. With the incident light in visible wavelength region, it was predicted that there might be three phenomena on the surface of coated leaves. One phenomenon was the light absorption by copper. Another phenomenon was the interference due to the reflection inside the nanostructure of leaves. The last phenomenon was the localized surface plasmon resonance, which caused greater absorption at the plate edges. In order to clarify the interaction between the copper layer and nanostructure on the leaves, the optical properties of the copper-coated leaves were calculated.

**Figure 6:**
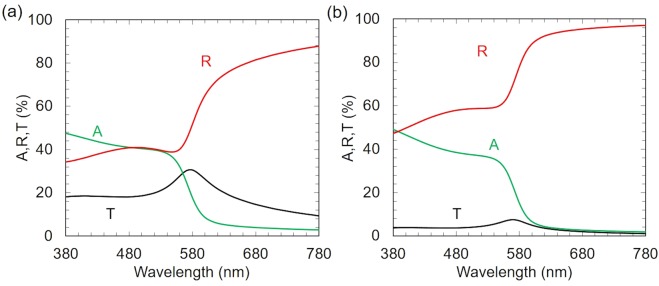
Calculated reflectance *R*, transmittance *T*, and absorption *A* spectra of (**a**) 25-nm-thick and (**b**) 50-nm-thick flat copper films.

Figure 6(a,b) show the calculated reflectance (R), transmittance (T) and absorption (A) spectra of flat copper thin films, which were 25 and 50 nm thick. The absorption A was calculated using the following equation A = 1 − T − R, because there was no scattering in flat copper surface. The calculation was done using the Transfer Matrix Method with refractive index data of copper from a previous study^22^. The reflectance (R) was more than 40% and 50% over visible spectral range even in cases of 25 nm and 50 nm-thick copper thin films, respectively. The absorption was about 40% and 3% in the wavelength ranges from 300 to 500 nm and from 580 to 780 nm, respectively. This explained the disappearance of peaks in reflectivity and scattering spectra of copper-coated *Phanera purpurea* and *Pistia stratiotes* leaves in the wavelength range from 500 to 560 nm. The absorption was weak (<5%) in the wavelength range of higher than 680 nm. Meanwhile, the reflectivity and scattering intensities of sputtered leaves were much lower than those of both non-sputtered leaves and copper thin films in this wavelength range. This implies that the considerably low reflectivity of the copper-coated leaves can be attributed to both copper layer and nanostructure in the leaves.

**Figure 7:**
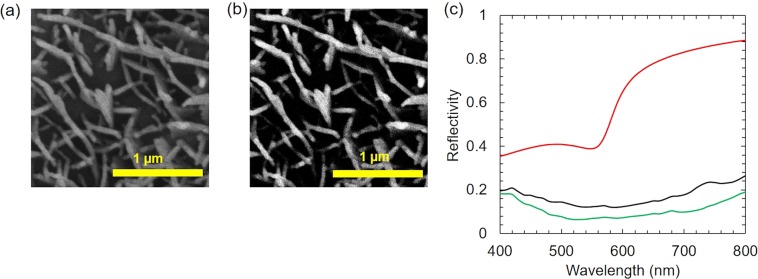
(**a**) SEM image for model calculation, (**b**) the binary-processed SEM image, (**c**) Calculated spectra of reflectivity.

The FDTD (FullWAVE simulation) calculation was employed to study the mechanism of the low reflectance of the copper-coated leave surface^16,17^. The calculation model was developed based on nanostructure on the leaf, which can be observed in the SEM images of the surface leaf. The SEM image and black-and-white binarized SEM image of *Pistia stratiotes* leaf are shown in Fig. 7(a,b), respectively. The black-and-white binarized SEM image showing the top-view image of the nanostructure on the investigated leaf was imported into the FDTD software. The structure of calculation model consisted of three layers. The top layer and the second layer were structured based on top view of the nanostructure from the binarized image. The top layer was a thick copper film on the nanostructure corresponding to the plate at the water cabbage leaf of surface. The 200 nm-thick dielectric materials with the refractive index of 1.5 was used as the second layer that was considered as the structure of leaf. This thickness was taken from the height of the plate that was determined from the SEM image. The bottom layer had two different areas including (i) the nanostructure area, which was dielectric materials and (ii) the thick copper film, which was deposited through the nanostructures in the actual leaf. The thickness of this layer was 30 nm. The space was divided into meshes of 5 nm × 5 nm × 5 nm and the perfectly matched layers (PML) were used for the boundary conditions.

The calculated spectra of reflectivity are shown in Fig. 7(c). The red line in Fig. 7(c) shows the reflectivity of 30 nm-thick copper thin films. The blue and black lines in Fig. 7(c) show the reflectivity of model leaf, when the thickness of the top layer was 30 and 50 nm, respectively. The reflectivity was 0.1–0.25 over the visible spectral range. The transmittance was predicted to be low. Thus, the low reflectivity was mainly due to the large absorption of the nanostructures. The reflectivity of a 30-nm and 50-nm thick copper thin films was higher than 0.75 at wavelengths longer than 600 nm, where the reflectivity of the purple bauhinia leaf model was much lower (0.15–0.25). Hence, these great differences may derive from the nanostructure of the leaf model. Accordingly, it is concluded that the observed low reflectivity originates from the nanostructures of the purple bauhinia leaf surface. The similar phenomena occurred with the *Phanera purpurea* leaf. There still exists the discrepancy between the observed (∼0.01) reflectivity and the calculated (0.1–0.25) reflectivity at the visible wavelengths. One reason is that it was impossible to replicate exactly the leaf nanostructures, as shown in the SEM images of Fig. 7(a) in the calculation model of leaves. The detailed calculations for determining the light behavior of these leaves using the FDTD are in progress and will be reported in the next papers.

## Conclusion

This study investigated three categories of leaves as the templates for fabrication of copper-based biometamaterials. The *Catharanthus roseu*s leaves represented the hydrophilic group, whereas *Pistia stratiotes* and *Phanera purpurea* leaves exemplified the hydrophobic group. The obtained results showed that nanostructures of *Pistia stratiotes* and *Phanera purpurea* leaves were random orientation and dense distribution. The leaves of these two plants after being coated with a copper layer of 25–100 nm demonstrated the great light absorbance (approximately 90%). Finite-difference time-domain (FDTD) calculations revealed that the interaction between leaf nanostructure and the copper coating layer resulted in a reduction in the reflection of the copper-coated leaf. This is probably regarded as a main reason for the enhancement in the light absorption of copper coated leaves. It is expected that this work can contribute to the fabrication of the black or light absorbing materials in an effort to mitigate the cost and simplify the preparation procedure. Development of high-performance solar steam generation based on light absorbing thin films will be reported in the next papers.

## Supplementary information

Supplementary Data.
