# Supplementary material for: Bio-inspired broadband absorbers induced by copper nanostructures on natural leaves
Source: Sci Rep. 2020 Feb 24;10:3243. doi: 10.1038/s41598-020-59960-x (PMC7039963; doi:10.1038/s41598-020-59960-x)
Supplement: Supplementary file 1 — Supplementary Data. [file 41598_2020_59960_MOESM1_ESM.pdf]

Supplementary Information

Corresponding author: Correspondence and requests for materials should be addressed to T. T. P.: pt.thanh@vju.ac.vn

Bio-inspired broadband absorbers induced by copper nanostructures on natural leaves

Trung Duc Dao[1], Dinh Dat Pham[1], Thi An Hang Nguyen[1], Thi Viet Ha Tran[1], Chung Vu Hoang[2], Tien Thanh Pham[1,*]

[1] VNU Vietnam – Japan University, Vietnam National University, Hanoi, Luu Huu Phuoc Street, My Dinh 1 Ward, Nam Tu Liem District, Hanoi 100000, Vietnam

[2] Institute of Materials Science, Vietnam Academy of Science and Technology, 18 Hoang Quoc Viet Street, Cau Giay District, Hanoi, Vietnam

The wetting performance of water cabbage and purple bauhinia leaves

Theoretically, a solid surface is considered as a hydrophobic or water repellent when the static water contact angle of water droplet on that surface higher than 90°. Water contact angle analysis was carried out in order to confirm the wetting performance of water cabbage and purple bauhinia leaves and the results were shown in Figure S1. The water contact angle was measured using a Smart drop contact angle system maintained by a computer-controlled device with the 3.9 μL droplet at room temperature (21.9°C). The results showed that the value of water contact angle on water cabbage and purple bauhinia are 120.8° and 119°, respectively. It is the evidence for the highly water repellence property of these two kinds of leaf.

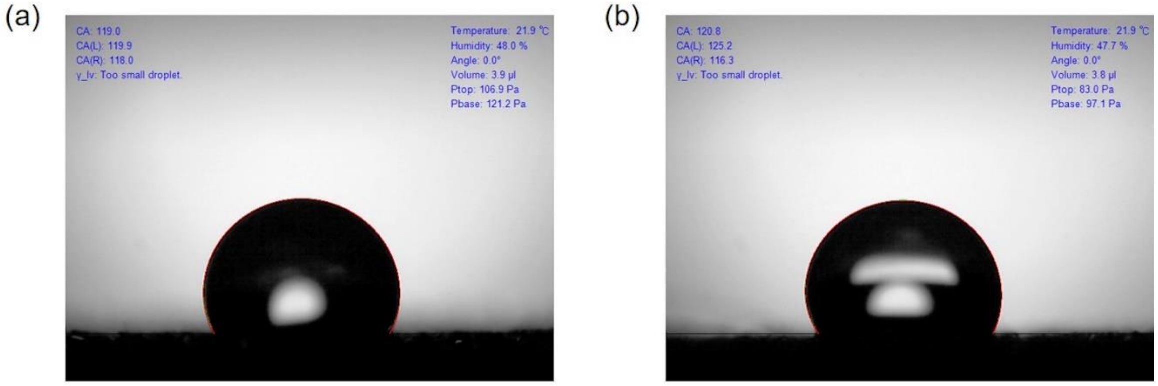

Figure S1. The wetting performance of water, (a) purple bauhinia, (b) water cabbage

The conditions of copper coating method

The sputtering was performed using a SKE106012 (Syskey Technology Co., Ltd,) sputtering coater. The sputtering was done with a DC power of 100 W and a total pressure of about 0.13 Pa of argon (99.99% purity). The deposition was conducted at the room temperature

with the deposition time ranging from 20 to 300s. In order to determine the thickness of copper coated layer, the copper films were also deposited onto glass substrates under the same conditions. The thickness of the copper layer on the surface of leaves was determined through the thickness of that on the glass substrates by measuring thin film step heights with the NanoMap 500LS. The thicknesses of copper layer are show in Figure S2 as a function of the deposition time.

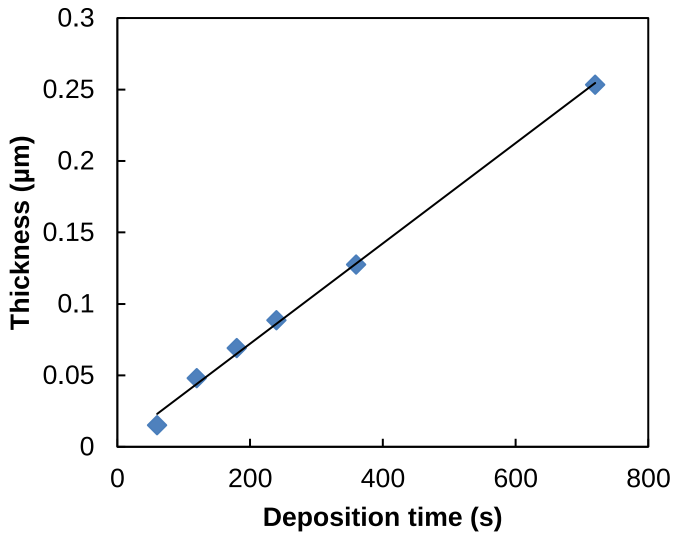

Figure S2. The thicknesses of copper layer

SEM images of three kinds of plants

The large multiple SEM images of water cabbage, purple bauhinia and Catharanthus roseus leaves are show in Figure S3.

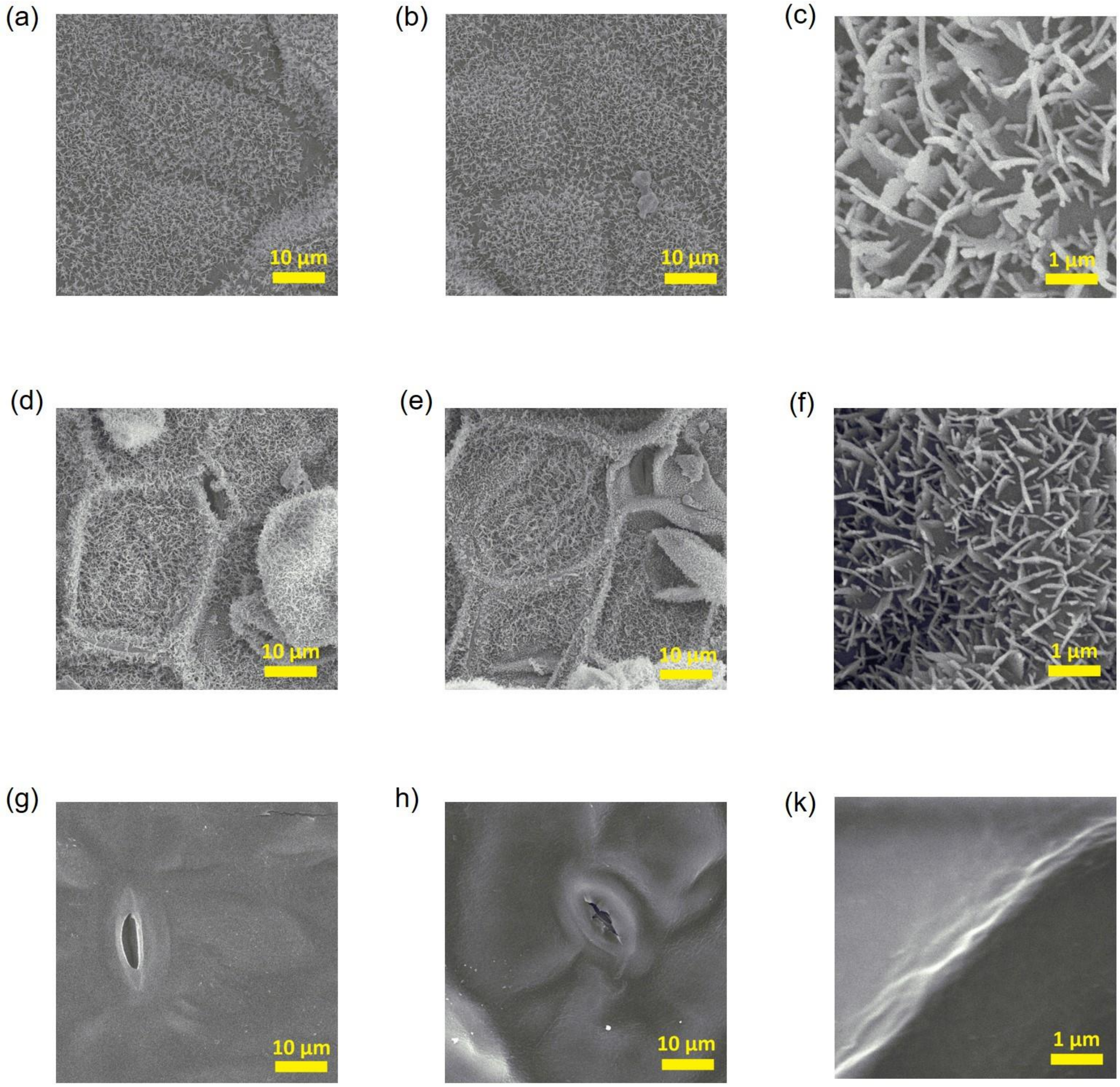

Figure S3. SEM images of copper-coated leaves: (a) (bar: 10 μm), (b) (bar: 10 μm), (c) (bar: 1 μm) magnified images of *Phanera pupurea* leaf, (d) (bar: 10 μm), (e) (bar: 10 μm), (f) (bar: 1 μm), magnified images of *Pistia stratiotes* leaf, (g) (bar: 10 μm), (h) (bar: 1 μm) magnified images of *Catharanthus roseus* leaf.
